# P246: Evolution of S. pneumoniae serotypes in invasive pneumococcal diseases (IPD) in Western Europe: a literature review

**DOI:** 10.1186/2047-2994-2-S1-P246

**Published:** 2013-06-20

**Authors:** D Christopoulou, M Tin Tin Htar, E Murray, E Bonnet, RR Reinert

**Affiliations:** 1 Pfizer, London, UK; 2 Pfizer, Paris, France; 3 MAPI Research Trust, London, UK

## Introduction

Pneumococcal diseases (PDs) are a major cause of morbidity and mortality worldwide. Pneumococcal conjugate 7-valent vaccine (PCV7) was introduced in national immunization programs (NIPs) in 2006 in many European countries; higher valency PCVs became available in 2009-2012.

## Objectives

To describe the incidence and evolution of serotype distribution of IPD in all ages.

## Methods

Medline, Embase, public health websites and conference abstracts were searched from January 2010-July 2012 for data on incidence and *S. pneumoniae* serotype distribution in all PDs and nasopharyngeal carriage in all ages in Western Europe. Here we present only the data for serotype distribution in IPDs between 2005-2011.

## Results

We identified 1431 sources; of the 124 included, 70 reported serotype data in children and adults with IPD from 15 countries. Before PCV introduction, PCV7 and PCV13 serotypes represented 53-77% and 91% of those found in children and 14-46% and 75% in adults, respectively. After PCV7 introduction this changed to 1-29% and 81-94% in children and 13-31% and 68-70% in adults. Before PCV introduction the most prevalent serotypes (≥5%) were 14, 19A, 1, 23F, 19F, 6B, 18C, 7F, 9V in children and 14, 3, 7F, 4, 5, 1, 9V, 19A, 23F, 6B in adults. After PCV7 introduction the most prevalent serotypes were 19A, 7F, 14, 3, 1, 6A, 18C, 5 in children and 7F, 19A, 1, 3, 15, 14, 4, in adults. The distributions differed across the countries.

## Conclusion

After PCV7 introduction, in both children and adults, the prevalence of PCV7 serotypes was reduced, while serotypes 1, 19A, 7F, 3 and 6A became relatively more prevalent. Newly available PCVs including more serotypes will be helpful in effective control of PDs caused by these serotypes. Continuous surveillance is necessary for better understanding of evolution of PDs in the PCV vaccination era.

## Disclosure of interest

D. Christopoulou Employee of Pfizer, M. Tin Tin Htar Employee of Pfizer, E. Murray Consultant for Pfizer, E. Bonnet Employee of Pfizer, R. R. Reinert Employee of Pfizer

